# Assessing Risk of Nephrolithiasis in Urology Residents: Are We Practicing What We Preach?

**DOI:** 10.7759/cureus.103483

**Published:** 2026-02-12

**Authors:** Hope Maine, Aashka Sheth, Craig Ziegler, Kellen Choi

**Affiliations:** 1 Department of Urology, University of Louisville School of Medicine, Louisville, USA; 2 Graduate Medical Education Research, University of Louisville School of Medicine, Louisville, USA; 3 Department of Urology, University of Louisville Hospital, Louisville, USA

**Keywords:** kidney stones, nephrolithiasis, nephrolithiasis risk factors, residency, urology

## Abstract

Purpose

Nephrolithiasis is a common urological condition that is treated by urologists. Urologists counsel patients on the importance of adequate hydration to decrease the risk of nephrolithiasis as well as the avoidance of specific diets that can contribute to the condition. To date, the risk of urology resident physicians developing nephrolithiasis during their residency has not been studied. This study aimed to investigate nephrolithiasis in urology residents and factors that may contribute to their potential protection or increased susceptibility to this pathology. Based on self-reported water intake, most respondents reported fluid consumption below commonly cited hydration targets, though guideline awareness and adherence were not directly assessed. Previous studies have examined residents’ satisfaction with their programs, but none have investigated the occurrence of kidney stones among residents.

Materials and methods

Through a nationally distributed survey of current urology residents, this pilot study aimed to address these questions by examining variables such as caffeine consumption, PGY year, family history of kidney stones, personal history of kidney stones, and life satisfaction.

Results

The results indicated that as urology residents' PGY level increased, their water intake decreased. In addition, life satisfaction increased as PGY increased.

Conclusion

Future research on this topic will aim to expand the study cohort to include attending physicians at a later stage in their careers to compare attitudes and actions that may contribute to the occurrence of nephrolithiasis.

## Introduction

Nephrolithiasis is a urological condition associated with decreased urine volume or increased concentrations of specific solutes, such as calcium, uric acid, cystine, xanthine, or phosphate [1]. These factors lead to the crystallization of solutes in urine and subsequent stone formation. Stones are precipitated by risk factors, such as low fluid intake, hot climate, personal or family history, and metabolic syndrome [1]. Additionally, stone formation is more prevalent in males and non-Hispanic white individuals. Renal damage, pyelonephritis, and obstructive nephropathy are complications of long-standing nephrolithiasis that can predispose individuals to chronic kidney disease [2]. Another complication of stones is their high recurrence rate, which is approximately 50% over the next 10 years [3]. To prevent stone formation, urologists recommend daily fluid intake to produce a urine output of 2.5 liters [4]. Typically, producing this amount of urine would mean intaking at least 2.5 L of fluid or more [5]. The type of fluids consumed is equally important, through large-volume water intake instead of the consumption of certain beverages, such as coffee, tea, and alcohol [6]. Additional factors, such as increased physical activity, decreased smoking, and daily calcium intake, are protective against the development of nephrolithiasis; however, more evidence is needed to determine the correlation between these variables [6].

Previous studies have looked at this question with regard to those who work in healthcare [7], but none so far have looked specifically at this question in urology residents. It has previously been reported that certain occupations, such as long-haul drivers, nurses, and operating room (OR) workers, are associated with kidney stone development due to the limited time allotted to use the restroom [8]. All of these occupations involve prolonged time with little access to restrooms, which can be seen in urology residency as well through factors like long operative times. With regard to urology residency specifically, there has been a study looking at resident wellness and specific wellness programs for residents [9], which can also add to our survey results and discussion. Urology residency is a demanding residency, with one study finding that 38-68% of residents reported feeling burnout [10]. Feeling burnt out could lead to one not prioritizing their own health. One of the variables associated with a significant effect on burnout was working more than an 80-hour week [10]. Stress and poor sleep quality have also been implicated in kidney stone formation, which are factors present in many medical residents [11,12]. Diet has also been implicated in the formation of kidney stones [3]. All of these risk factors suggest that healthcare workers are at an increased risk for kidney stone formation, and this study sought to address whether urology residents are also at risk or whether their knowledge on this specific topic serves as a protective factor for kidney stone formation.

Nephrolithiasis is a frequently encountered pathology of the urinary system [1]. It is a condition urology residents will undoubtedly be treating throughout their training and for the rest of their careers, as the prevalence is also increasing [6]. Therefore, this study sought to establish whether this worldwide occurrence of nephrolithiasis of 12% is also found among physicians who are likely to treat this condition [13]. There are also other variables that can be studied within this population to add more literature and evidence to the potential (or proven) causative agents of nephrolithiasis through variables such as stress level [11,14] and lack of fluid consumption [15]. Another variable that can be studied is the correlation between caffeine content and nephrolithiasis, as other studies have found a negative association between the two variables [16,17] and that there is a protective effect of caffeine use [18]. Additionally, this study can provide insights into how urology resident satisfaction relates to wellness, especially as residents progress throughout their careers. There have been no other studies looking at this direct topic, as well as similar topics such as how much water residents in general drink daily, although there have been multiple studies on different areas of resident satisfaction among different specialties [19-21].

Another area we were interested in was whether family history of kidney stones influenced the amount of fluid intake by residents. Multiple studies have shown that there is a genetic influence on kidney stone development [22,23]. We wanted to see if this would be a trend, potentially impacting urology residents as well.

## Materials and methods

The final sample size for this pilot study included 26 respondents (n=26). The data were collected from current urology residents across the nation and de-identified. There were no inclusion or exclusion criteria based on age, sex, childbearing potential, or racial/ethnic origins. No interventions were performed in this study.

A survey, newly developed as a pilot study, was created with the intention of being very quick to complete (see Appendix) to gather information on the variables we wanted to study, including caffeine consumption, PGY year, family history of kidney stones, personal history of kidney stones, and life satisfaction. Surveys were emailed to urology programs across the country, and the link to the survey was posted on various social media accounts.

The statistical methods used included frequency and cross-tabulation tables presenting counts and percentages. Exact tests, i.e., Fisher’s Exact Test and the Fisher-Freeman-Halton Exact Test, were used to initially assess statistically significant associations between the categorical variables. Exact test methods are well-suited for smaller sample sizes, such as in this study (n = 26). No significant associations were found, which may be due to low statistical power. Due to the ordinal measurement of several variables and the small sample size, Spearman’s correlations were used in a secondary analysis to explore relationships between select ordinal variables. Specifically, PGY year was correlated with the amount of water drank daily, the type of caffeinated beverage consumed most, the amount of caffeine consumed daily, satisfaction with current life, and times per week feeling mentally exhausted. Spearman’s correlations maintain the inherent ranking from ordinal data and often have increased power over nominal-based exact tests that ignore the rank information of these scales. Correlation tables with p-values and cross-tabulation data are included in the Results. Statistical significance was set at p < .05, and all tests were 2-tailed. SPSS for Windows Version 30 (IBM Corp., Armonk, NY, USA) was used to analyze the data.

## Results

In terms of demographics, the largest group of respondents were PGY-5 residents, making up 26.9% of respondents. PGY-1 and PGY-2 were the second largest groups of respondents, accounting for 23.1% of the respondents. Women comprised the majority of the study group (57.7%). The largest age group was 25-30-year-olds, making up 57.7% of respondents. By postgraduate year (PGY), most respondents drank coffee for their caffeine usage, with the exception of PGY-3, which was split 50/50 by coffee or no caffeine use. Of all the participants, 65% reported coffee as their most consumed beverage. PGY-5 residents reported the highest caffeine usage, with 42.9% drinking 200+ mg daily. The most common responses for average caffeine consumption were 200 mg/day and 96-150 mg/day for PGY-1 residents, 151-200 mg/day for PGY-2 residents, and 0 mg and 46-96 mg/day for PGY-3 residents. The responses were evenly split among the PGY-4 residents.

Seven out of 26 (about 26.9%) respondents reportedly drank more than 1 liter of water per day. Only one respondent (PGY-1) drank 3+ liters. The most common responses for average daily water intake were 1.1-2 liters for PGY-1 residents, 1 liter for PGY-2 residents, 16 oz.-33 oz. for PGY-3 residents, and 1 liter for PGY-4 residents. The majority of PGY-5 residents reported between 16 oz.-33 oz. (42.9%) daily. The most common response among all respondents was about 1 liter per day. None of the residents had a history of kidney stones; however, six had a family history. For those with a family history of nephrolithiasis, the majority of respondents (50%) reported drinking 16-33 oz. of water daily. Those with no family history had a majority group of approximately 40% of respondents drinking approximately 1 liter of water daily.

In terms of mental exhaustion, 11.5% of respondents reported never feeling mentally exhausted during a week, 23% reported 1-2 times a week, and 26.9% reported 3-4 times a week; 38.5% of respondents reported feeling mentally exhausted more than five times per week. There was no clear trend in PGY and mental exhaustion.

The respondents’ demographic data are presented in Table 1. Two statistically significant associations were identified; however, these findings should be interpreted cautiously given the small sample size and exploratory nature of the analyses (Table 2). None of the other data collected, as shown in Tables 3-5, was found to be statistically significant.

**Table 1:** Demographic Variables

PGY Year, N (%)	PGY1	6	(23.1%)
	PGY2	6	(23.1%)
	PGY3	2	(7.7%)
	PGY4	5	(19.2%)
	PGY5	7	(26.9%)
Gender, N (%)	Woman	15	(57.7%)
	Man	11	(42.3%)
Age (in years), N (%)	25-30	15	(57.7%)
	31-36	9	(34.6%)
	37-42	2	(7.7%)

**Table 2:** Spearman’s Correlation of Select Urological Guidelines and Wellness Variables by PGY Years

		On average, how much water do you drink throughout the day?	What type of caffeinated beverages do you consume the most?	On average, how many milligrams of caffeine do you consume per day?	How satisfied do you feel with your life currently?	How many times per week do you feel mentally exhausted?
PGY Year	Spearman’s R	-.536	-.058	.122	.431	-.033
	p-value	.005	.783	.551	.032	.873
	N	26	25	26	25	26

**Table 3:** Frequency and Percentages of Select Urological Guidelines and Wellness Variables

What type of caffeinated beverages do you consume the most?, N (%)	None	4	(15.4%)
	Tea (~45 mg caffeine/cup)	1	(3.8%)
	Coffee (~95 mg caffeine/cup)	17	(65.4%)
	Energy drinks (~150-200 mg/serving)	3	(11.5%)
	Coke zero	1	(3.8%)
On average, how many milligrams of caffeine do you consume per day?, N (%)	0 mg	3	(11.5%)
	1-45 mg	4	(15.4%)
	46-95 mg	3	(11.5%)
	96-150 mg	6	(23.1%)
	151-200 mg	4	(15.4%)
	200+ mg	6	(23.1%)
On average, how much water do you drink throughout the day?, N (%)	8 oz. (1 glass)	3	(11.5%)
	16 oz.-33 oz.	7	(26.9%)
	33 oz. (about 1 liter)	9	(34.6%)
	1.1 liters-2 liters	6	(23.1%)
	3+ liters	1	(3.8%)
Have you experienced kidney stones since beginning residency? N, (%)	No	26	(100%)
	Yes	0	(0.0%)
Family history of kidney stones?, N (%)	Yes	6	(23.1%)
	No	20	(76.9%)
How many times per week do you feel mentally exhausted?, N (%)	Never	3	(11.5%)
	1-2 times	6	(23.1%)
	3-4	7	(26.9%)
	5+	10	(38.5%)
How satisfied do you feel with your life currently?, N (%)	Very dissatisfied	0	(0.0%)
	Dissatisfied	1	(3.8%)
	Somewhat dissatisfied	2	(7.7%)
	Neither satisfied nor dissatisfied	2	(7.7%)
	Somewhat satisfied	5	(19.2%)
	Satisfied	10	(38.5%)
	Very satisfied	5	(19.2%)
	No response	1	(3.8%)

**Table 4:** Cross-Tabulation of Select Urological Guidelines and Wellness Variables by PGY Years

		PGY1		PGY2		PGY3		PGY4		PGY5		
		N	(%)	N	(%)	N	(%)	N	(%)	N	(%)	P-Value
What type of caffeinated beverages do you consume the most?	None	1	(16.7%)	1	(16.7%)	1	(50.0%)	1	(20.0%)	0	(0.0%)	.261
	Tea (~45 mg caffeine/cup)	0	(0.0%)	0	(0.0%)	0	(0.0%)	1	(20.0%)	0	(0.0%)	
	Coffee (~95 mg caffeine/cup)	3	(50.0%)	4	(66.7%)	1	(50.0%)	2	(40.0%)	7	(100.0%)	
	Energy drinks (~150-200 mg/serving)	2	(33.3%)	0	(0.0%)	0	(0.0%)	1	(20.0%)	0	(0.0%)	
	Coke zero	0	(0.0%)	1	(16.7%)	0	(0.0%)	0	(0.0%)	0	(0.0%)	
On average, how many milligrams of caffeine do you consume per day?	0 mg	1	(16.7%)	0	(0.0%)	1	(50.0%)	1	(20.0%)	0	(0.0%)	.390
	1-45 mg	0	(0.0%)	2	(33.3%)	0	(0.0%)	1	(20.0%)	1	(14.3%)	
	46-95 mg	1	(16.7%)	0	(0.0%)	1	(50.0%)	1	(20.0%)	0	(0.0%)	
	96-150 mg	2	(33.3%)	1	(16.7%)	0	(0.0%)	1	(20.0%)	2	(28.6%)	
	151-200 mg	0	(0.0%)	3	(50.0%)	0	(0.0%)	0	(0.0%)	1	(14.3%)	
	200+ mg	2	(33.3%)	0	(0.0%)	0	(0.0%)	1	(20.0%)	3	(42.9%)	
On average, how much water do you drink throughout the day?	8 oz. (1 glass)	0	(0.0%)	0	(0.0%)	0	(0.0%)	1	(20.0%)	2	(28.6%)	.122
	16 oz.-33 oz.	1	(16.7%)	0	(0.0%)	2	(100.0%)	1	(20.0%)	3	(42.9%)	
	33 oz. (about 1 liter)	1	(16.7%)	5	(83.3% )	0	(0.0%)	2	(40.0%)	1	(14.3%)	
	1.1 liters-2 liters	3	(50.0%)	1	(16.7%)	0	(0.0%)	1	(20.0%)	1	(14.3%)	
	3+ liters	1	(16.7%)	0	(0.0%)	0	(0.0%)	0	(0.0%)	0	(0.0%)	
Family history of kidney stones?	Yes	0	(0.0%)	1	(16.7%)	1	(50.0%)	1	(20.0%)	3	(42.9%)	.366
	No	6	(100.0%)	5	(83.3%)	1	(50.0%)	4	(80.0%)	4	(57.1%)	
How many times per week do you feel mentally exhausted?	Never	0	(0.0%)	2	(33.3%)	0	(0.0%)	0	(0.0%)	1	(14.3%)	.832
	1-2 times	2	(33.3%)	0	(0.0%)	1	(50.0%)	2	(40.0%)	1	(14.3%)	
	3-4	2	(33.3%)	1	(16.7%)	0	(0.0%)	1	(20.0%)	3	(42.9%)	
	5+	2	(33.3%)	3	(50.0%)	1	(50.0%)	2	(40.0%)	2	(28.6%)	

**Table 5:** Cross-Tabulation of Select Urological Guidelines and Wellness Variables by Family History of Kidney Stones

		Family history of kidney stones?				
		Yes		No		
		N	(%)	N	(%)	P-Value
What type of caffeinated beverages do you consume the most?	None	2	(33.3%)	2	(10.0%)	.688
	Tea (~45 mg caffeine/cup)	0	(0.0%)	1	(5.0%)	
	Coffee (~95 mg caffeine/cup)	4	(66.7%)	13	(65.0%)	
	Energy drinks (~150-200 mg/serving)	0	(0.0%)	3	(15.0%)	
	Coke zero	0	(0.0%)	1	(5.0%)	
On average, how many milligrams of caffeine do you consume per day?	0 mg	1	(16.7%)	2	(10.0%)	.507
	1-45 mg	2	(33.3%)	2	(10.0%)	
	46-95 mg	1	(16.7%)	2	(10.0%)	
	96-150 mg	0	(0.0%)	6	(30.0%)	
	151-200 mg	1	(16.7%)	3	(15.0%)	
	200+ mg	1	(16.7%)	5	(25.0%)	
On average, how much water do you drink throughout the day?	8 oz. (1 glass)	1	(16.7%)	2	(10.0%)	.642
	16 oz.-33 oz.	3	(50.0%)	4	(20.0%)	
	33 oz. (about 1 liter)	1	(16.7%)	8	(40.0%)	
	1.1 liters-2 liters	1	(16.7%)	5	(25.0%)	
	3+ liters	0	(0.0%)	1	(5.0%)	
How many times per week do you feel mentally exhausted?	Never	0	(0.0%)	3	(15.0%)	
	1-2 times	1	(16.7%)	5	(25.0%)	
	3-4	2	(33.3%)	5	(25.0%)	
	5+	3	(50.0%)	7	(35.0%)	

## Discussion

This study provides preliminary, hypothesis-generating data regarding hydration behaviors among urology residents and highlights areas for future research rather than direct implications for population-level nephrolithiasis management. We studied the behaviors of residents to assess their risk for nephrolithiasis. This would ultimately lead to fewer emergency department visits for this condition and place less burden on the healthcare system. In addition, it could illuminate some of the problems that residents face during residency, which could facilitate conversations about how to alleviate some of the possible potentiating factors leading to a higher occurrence of nephrolithiasis. Overall, this could improve their working conditions and help them remain as healthy as possible during their residency.

Based on our results, it appears that those with a family history of nephrolithiasis do not change their drinking habits. However, these results were from a small sample size and would be better evaluated with a larger cohort. We hypothesized that a family history could affect their drinking habits, with them intaking more water than those without a family history.

For our statistically significant results, we hypothesized that as PGY increases, there is a possibility that residents have less time to hydrate, as they are in the OR more and in longer cases. One possible explanation for the observed association is that higher PGY levels may be accompanied by increased responsibilities and operative time; however, this relationship was not directly measured in this study. In terms of life satisfaction and the statistically significant result we saw with improvement as PGY increased, we hypothesize that this could be due to being in the operating room more and being able to have more autonomy and be more in charge of their plans and patient care. In addition, they are getting closer to practicing on their own, which would likely be highly satisfactory.

Our study found that 96.1% of urology residents did not meet the daily fluid intake recommended by urologists, as only one participant reported drinking 2.5 L of water or more daily. There could be numerous reasons why residents do not drink the amount of water set by urological associations, as well as not changing habits based on family history, but the largest variable is likely time constraints. Residents are busy and likely do not focus on their daily water intake, as they have many other responsibilities to take care of. Previous studies assessing resident well-being have shown that medical residents have suboptimal diet quality, and it is likely that if residents are unable to make time for proper nutrition, they also have inadequate water intake [24,25]. If this is the case, this is an important factor that residency programs should be aware of, as resident health and well-being should be prioritized. Another reason could be that, especially in long cases, being very hydrated would cause problems for them, as they would not be able to scrub out to go to the bathroom. Another potential reason is that much of their liquid intake is caffeine, so they do not add more liquid through water during the day.

As this study had a small sample size of 26 participants, this is a limitation of our study, which likely impacted the power and, therefore, the statistical significance. It is also limited in generalizability due to the small sample size. Other limitations include this being self-reported data, a cross-sectional study, and non-response bias due to the survey aspect of data collection. As the study was sent to program coordinators and posted on social media, there could also be some residents who did not see the survey and were therefore not involved in it.

## Conclusions

Our study aimed to address several questions regarding urology residents and the prevalence of nephrolithiasis, including whether residents follow guidelines established by urologic associations and whether family history influences water intake and hydration habits. Although we did not find statistical significance with these questions, we identified possible reasons for these findings. We observed statistical significance among other variables, suggesting that the demands of residency are likely to take a toll on the amount of water residents can drink daily. Overall, this novel study addresses a question that has not yet been explored and sparks discussion about the reality of staying hydrated for residents, the impact of hydration on life satisfaction during residency, and potential areas for further investigation in future studies involving residents, nephrolithiasis, and water-drinking guidelines set by urological associations. Future studies with larger sample sizes and broader populations, including attending physicians and other urologists with longer practice experience, would be beneficial.

## Appendices

1. What year are you in your urology residency?

a. PGY1

b. PGY2

c. PGY3

d. PGY4

e. PGY5

2. Have you experienced kidney stones since beginning residency?

a. Yes

b. No

3. Which most closely describes your gender?

a. Woman

b. Man

c. Non-binary

d. Prefer not to state

e. Gender not listed. My gender is…_______

4. What is your age?

a. 25-30 years old

b. 31-36 years old

c. 37-42 years old

d. 43-49 years old

e. 50 + years old

5. What type of caffeinated beverages do you consume the most?

a. Coffee (~95 mg caffeine/cup)

b. Tea (~45 mg caffeine/cup)

c. Energy drinks (~150-200 mg/serving)

d. Other (please specify including the average amount of caffeine in a drink): ______

6. On average, how many milligrams of caffeine do you consume per day?

a. 0 mg

b. 1-45 mg

c. 46-95 mg

d. 96-150 mg

e. 151-200 mg

f.  200+ mg

7. On average, how much water do you drink throughout the day?

a. 8 oz.

b. 16 oz.-33 oz.

c. 33 oz. (about 1 liter)

d. 1.1 liters-2 liters

e. 2.1 liters-3 liters

f. 3+ liters

8. Family history of kidney stones?

a. Yes

b.  No

9. How many times per week do you feel mentally exhausted?

a. Never

b. 1-2 times

c. 3-4 times

d. 5+ times

10. How satisfied do you feel with your life currently?

a. Very satisfied

b. Satisfied

c. Somewhat satisfied

d. Neither satisfied nor dissatisfied

e. Somewhat dissatisfied

f. Dissatisfied

g. Very dissatisfied
